# A New Member of Gamma-Conotoxin Family Isolated from *Conus princeps* Displays a Novel Molecular Target

**DOI:** 10.3390/toxins8020039

**Published:** 2016-02-05

**Authors:** Johanna Bernáldez, Samanta Jiménez, Luis Javier González, Jesús Noda Ferro, Enrique Soto, Emilio Salceda, Daniela Chávez, Manuel B. Aguilar, Alexei Licea-Navarro

**Affiliations:** 1 Departamento de Innovación Biomédica, CICESE, Carretera Ensenada-Tijuana 3918, Ensenada, Baja California, C.P. 22860, Mexico; jbernald@cicese.edu.mx (J.B.); mjimenez@cicese.edu.mx (S.J.); silem@cicese.edu.mx (D.C.); 2 Laboratorio de Espectrometría de Masas, Departamento de Proteómica, Centro de Ingeniería Genética y Biotecnología, Avenida 31 e/158 y 190, Cubanacán, Playa, PO Box 6162. C.P. 10600, La Habana, Cuba; luis.javier@cigb.edu.cu (L.J.G.); jesus.noda@cigb.edu.cu (J.N.F.); 3 Instituto de Fisiología, Benemerita Universidad de Puebla, 14 sur 6301, CU, San Manuel, Puebla, Pue, C.P. 72570, Mexico; esoto24@gmail.com (E.S.); emilio.salceda@gmail.com (E.S.); 4 Laboratorio de Neurofarmacología Marina, Departamento de Neurobiología Celular y Molecular, UNAM, Juriquilla, Queretaro, C.P. 76230, Mexico; maguilar@unam.mx

**Keywords:** *Conus princeps*, calcium channel, gamma-conotoxin, mass spectrometry, dorsal root ganglion neurons

## Abstract

A novel conotoxin, named as PiVIIA, was isolated from the venom of *Conus princeps*, a marine predatory cone snail collected in the Pacific Southern Coast of Mexico. Chymotryptic digest of the S-alkylated peptide in combination with liquid chromatography coupled to tandem mass spectrometry, were used to define the sequencing of this peptide. Eleven N-terminal amino acids were verified by automated Edman degradation. PiVIIA is a 25-mer peptide (CDAOTHYCTNYWγCCSGYCγHSHCW) with six cysteine residues forming three disulphide bonds, a hydroxyproline (O) and two gamma carboxyglutamic acid (γ) residues. Based on the arrangement of six Cys residues (C-C-CC-C-C), this conotoxin might belong to the O2-superfamily. Moreover, PiVIIA has a conserved motif (-γCCS-) that characterizes γ-conotoxins from molluscivorous *Conus*. Peptide PiVIIA has 45% sequence identity with γ-PnVIIA—the prototype of this family. Biological activity of PiVIIA was assessed by voltage-clamp recording in rat dorsal root ganglion neurons. Perfusion of PiVIIA in the µM range produces a significant increase in the Ca^2+^ currents, without significantly modifying the Na^+^, K^+^ or proton-gated acid sensing ionic currents. These results indicate that PiVIIA is a new conotoxin whose activity deserves further studies to define its potential use as a positive modulator of neuronal activity.

## 1. Introduction

Conventionally, conotoxins are classified into pharmacological families depending on the type of molecular target and the effect of the toxin upon it. Within conotoxins, well-known pharmacological targets are voltage-gated ion channels (Na^+^, K^+^, Ca^2+^), ligand-gated ion channels (nAChR, 5-HT_3_R), G-protein-coupled receptors (α1 adrenergic) and neurotransmitter transporters (noradrenaline). Regarding their action and specific targets, most of the conotoxin families have been characterized, and some of them, have attracted interest as drugs leads in the development of novel analgesics [1].

Well-characterized conotoxins are members of the O1-superfamily, which comprise δ-, μ- Voltage Gated Sodium Channel (VGSC) blockers, AnseAnsκ-Voltage Gated Potassium Channel (VGPC) blockers and ω-conotoxins Voltage Gated Calcium Channel (VGCC) blockers [2]; two examples are κ-PVIIA, proposed as a cardioprotectant agent, and ω-MVIIA (ziconotide) currently in clinical use for treatment of chronic pain [3,4]. The opposite is the case for O2-superfamily, it is one of the less thoroughly investigated group of conotoxins. The O2-superfamily includes conotoxins with cysteine frameworks VI and VII, and some have shown specific activity to mollusks [2].

Two O2-conotoxins, γ-TxVIIA and γ-PnVIIA isolated from the molluscivorous species of cone snails *C. textile* and *C. pennaceus*, respectively, share approximately 48% amino acid identity and 63% similarity. These peptides induce depolarization and increase firing of action potentials in molluscan neuronal systems. γ-TxVIIA produce strong excitatory effects specifically in neurons of *Aplysia* (a marine mollusk) but has no effect in other molluscan neuronal systems such as the caudodorsal neurons of *Lymnaea.* γ-PnVIIA triggers action potential bursting in isolated caudodorsal neurons from *Lymnaea* acting as an agonist of neuronal pacemaker cation currents [5,6]. These peptides have been classified as γ-conotoxins, and are the prototype of this pharmacological family of agonists of neuronal pacemaker cation currents.

In this work, we report a novel conotoxin named PiVIIA, isolated from venom duct of *Conus princeps*, a worm-hunting species collected in the Pacific Southern Coast of Mexico. Conotoxin PiVIIA contains one hydroxyproline and two gamma carboxyglutamic acids (γ) residues, where one of them is included in a conserved motif (-γCCS-), only found in the γ-conotoxin family. Based on the arrangement of the six Cys residues (C-C-CC-C-C), peptide PiVIIA might belong to the O2-superfamily. Surprisingly, it selectively increased the Ca^2+^ currents from dorsal root ganglion (DRG) neurons. Further studies are required to extend the characterization of PiVIIA and to define its role in pacemaker related channels. Nevertheless, this novel toxin expands our visions of γ-conotoxins and their potential molecular targets.

## 2. Results

### 2.1. Purification of the Major Native Peptide

**Figure 1:**
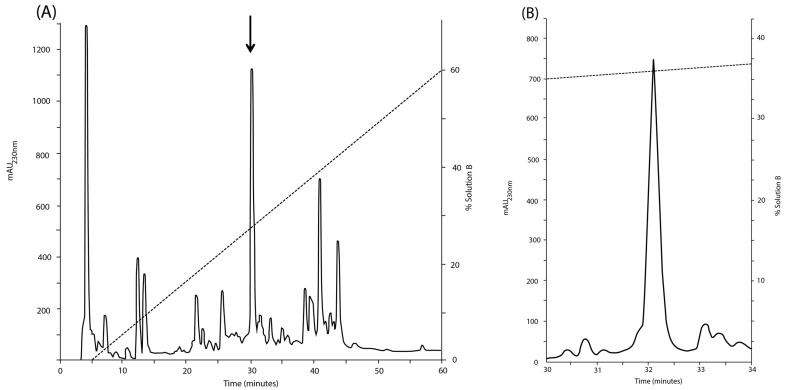
Purification of the venom extract of *Conus princeps*. (**A**) First fractionation step of the venom extract from *C. princeps* by using reverse phase-high performance liquid chromatography (RP-HPLC). The peptides were injected into a C_18_ column and eluted using a linear gradient of acetonitrile (ACN) solution containing 0.12% of trifluoroacetic acid (TFA) (*v*/*v*), keeping constant the flow rate at 1.09 mL·min^−1^, over 60 minutes. To obtain the pure PiVIIA peptide, the fraction indicated with an arrow in (**A**) was further purified in (**B**) with the same analytical C_18_ column using a linear gradient from 20% to 40% (*v*/*v*) at a flow rate of 0.5 mL·min^−1^, over 40 min. All purification protocols were conducted at room temperature and the elution profile was monitored at 230 nm. The broken lines indicate the increasing of the mobile phase.

From the crude venom of *C. princeps*, the upper half of the most prominent peak, which represents 12.07% of the total venom components determined by its relative peak area (Figure 1A), was selected for further purification. Its main component was eluted in apparently homogeneous form (Figure 1B). The purity and molecular mass were assessed by Liquid Chromatography-Mass Spectrometry (LC-MS) analysis, and confirmed by the detection of only one phenylthiohydantoin-derivative at every cycle of automated Edman degradation.

### 2.2. Molecular Mass of the Purified Peptide

**Figure 2:**
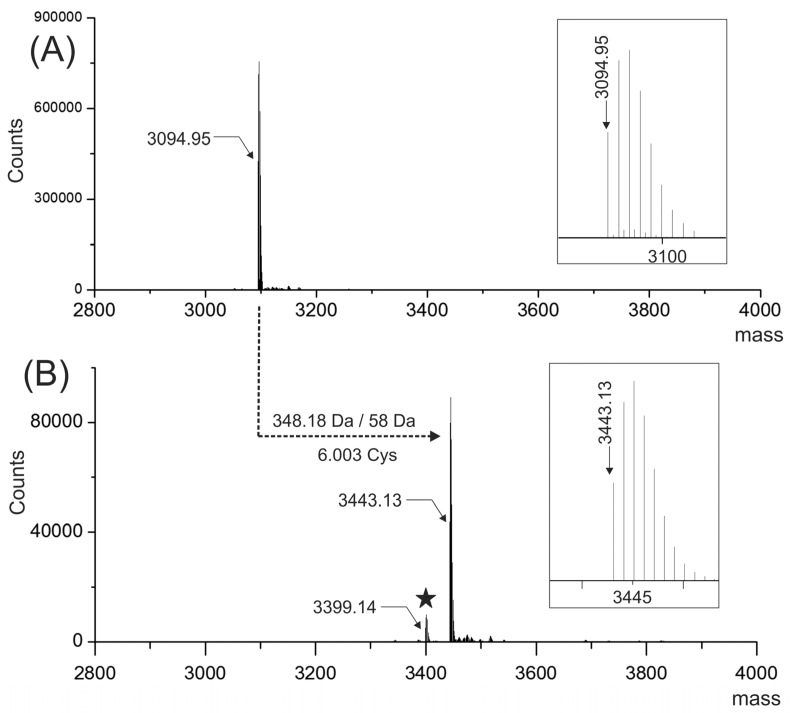
Deconvoluted ESI-MS spectra of the native (**A**) and the reduced carbamidomethylated (**B**) PiVIIA peptide. The insets shown in (**A**) and (**B**) correspond to the experimental isotopic ion distribution of the native and carbamidomethylated PiVIIA peptide, respectively. The molecular masses shown in this figure are expressed for the neutral molecule (M) and correspond to the monoisotopic peak in the isotopic ion distribution (see insets in (**A**) and (**B**)). The broken lines indicate the mass difference (348.18 Da) between the monoisotopic masses of signals shown in (**A**) and (**B**). The solid star shown in (**B**) highlights a signal with a molecular mass 43.99 Da lower than the carbamidomethylated peptide, presumably originated by an in-source fragmentation process tentatively assigned to decarboxylation.

Electrospray ionization-mass spectrometry (ESI-MS) analysis of the native peptide yield a mass spectrum with two intense 3+ and 2+ signals detected at *m/z* 1032.65 and 1548.48, respectively (data not shown). The ESI-MS spectrum was deconvoluted (Figure 2A), and it showed an intense signal with a molecular mass of 3094.95 Da (monoisotopic mass).

The native peptide was reduced and S-alkylated with iodoacetamide to elucidate the number of cysteine residues, and to facilitate the peptide sequencing by mass spectrometry. The ESI-MS spectrum of the reduced and carbamidomethylated peptide (Figure 2B) showed an intense monoisotopic signal with a molecular mass of 3443.13 Da. The mass increment of 348.18 Da between the signals shown in Figure 2A,B corresponds to the carbamidomethylation of six cysteine residues that were previously linked by three S-S bonds.

In Figure 2B, a signal with a molecular mass of 3399.14 Da (labeled with a solid star) is separated by 43.99 Da with respect to the signal of the reduced and carbamidomethylated peptide. This signal was assigned tentatively to a decarboxylation probably due to an in-source fragmentation process suggesting the presence of gamma carboxyglutamic acid, a labile post-translational modification [7,8].

### 2.3. Amino Acid Sequence of the Purified Peptide

**Figure 3:**
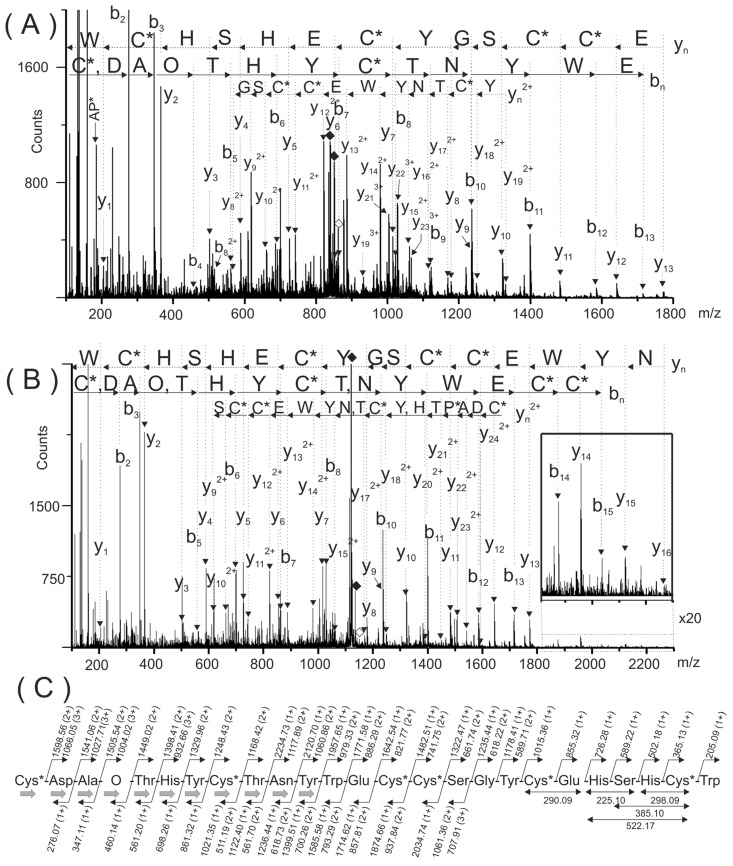
Electrospray ionization tandem mass spectrometry (ESI-MS/MS) spectra of the multiply-charged species (M + 4H)^4+^ and (M + 3H)^3+^ of the reduced and carbamidomethylated PiVIIA peptide detected at *m/z* 861.79 (**A**) and 1148.65 (**B**), respectively. The sequences deduced form the b*_n_* and y*_n_* ions are indicated with continuous and broken arrows, respectively. (**C**) Summary of the sequencing of peptide PiVIIA. The gray arrows in (**C**) indicated the eleven amino acids verified by stepwise Edman degradation. The solid and empty rhombuses indicate the precursor ion and neutral losses of CO_2_ molecules, respectively. O represents a hydroxyproline residue. The six cysteine residues are carbamidomethylated and are represented as C*.

ESI-MS analysis of the reduced and S-alkylated peptide yielded a mass spectrum with two intense signals at *m/z* 861.79 (4+) and 1148.65 (3+), respectively (data not shown). Both multiply-charged ions were fragmented by collision induced dissociation (CID) and the tandem mass spectrometry (MS/MS) spectra are shown in Figure 3A,B. Manual interpretation of the MS/MS spectra rendered a 25-mer peptide, ^1^CDAOTHYCTNYWECCSGYCEHSHCW^25^ (where O, denotes hydroxyproline).

The sequences deduced form the C- (y*_n_*^+^, y*_n_*^2+^ and y*_n_*^3+^) and N-terminal ions (b*_n_*) were highly complementary and yielded a very trustworthy sequencing. Immonium and internal ions are in a good agreement with the proposed sequence. Figure 3C summarizes the molecular masses of the backbone and some of the internal fragment ions used to deduce the amino acid sequence of the purified peptide. Due to the arrangement of Cys residues in the purified peptide (C-C-CC-C-C, where “-” denotes one or more non-Cys residues; framework VI/VII), it was named as PiVIIA, according to the current nomenclature for conotoxins [9].

Taking into account the mass accuracy of this measurement (0.01–0.02 Da mass error), the 113.04 Da mass differences between backbone ions y_22_^2+^ (*m/z* 1505.54) and y_21_^2+^ (*m/z* 1449.02) was assigned to hydroxyproline residue (O, 113.0477 Da) and not to Leu/Ile (113.0841 Da). The mass difference (*m/z* 113.03) between b_4_ (*m/z* 460.14) and b_3_ (*m/z* 347.11) ions confirmed this assignment. Other internal fragment ions were also in a good agreement with the assignment of this modified amino acid.

In addition, the automated Edman sequencing of the native peptide allowed the verification of the eleven N-terminal amino acids including the presence of hydroxyproline residue at the fourth position (see the black solid arrows in Figure 3C). During this analysis, no PTH-derivatives were detected at positions 1 and 8, and they were tentatively assumed to be Cys residues, which agrees with the sequence deduced from the results of *de novo* sequencing of the reduced and carbamidomethylated peptide.

However, the molecular mass (3006.96 Da, monoisotopic mass) of the sequence deduced from the ESI-MS/MS spectra (Figure 3A,B), considering three disulfide bonds, a hydroxyl proline residue and a free C-terminus is 87.99 Da lower than the experimental molecular mass (3094.95 Da) (Figure 2A).

This mass difference cannot be assigned to any of the 20 amino acids commonly present in proteins. In the region close to the [M+4H]^4+^ (*m/z* 861.79) and [M+3H]^3+^ (*m/z* 1148.65) precursor ions, two signals separated by 44 Da (see solid rhombuses in Figure 3A (*m/z* 850.79 and 839.79) and Figure 3B (*m/z* 1134.05 and 1119.39) were detected. These signals were tentatively assigned to the losses of two molecules of carbon dioxide, which is in agreement with the putative decarboxylation observed in Figure 2B (signal at 3399.14 Da). This evidence suggested that two labile posttranslational modifications (PTM) were lost upon CID conditions. As a consequence, two gamma-carboxy glutamic acids at positions 13 and 20 were assigned. In the MS/MS spectra (Figure 3A,B), the glutamic acids were detected in an unmodified form due to the poor stability of this PTM inside the collision cell. If the presence of two gamma-carboxy glutamic acids are considered beside the hydroxyproline residue, the calculated (3443.12 Da) and experimental monoisotopic mass (3443.13 Da) of the carbamidomethylated peptide are in a good agreement with a mass error of 0.01 Da (2.9 ppm).

**Figure 4:**
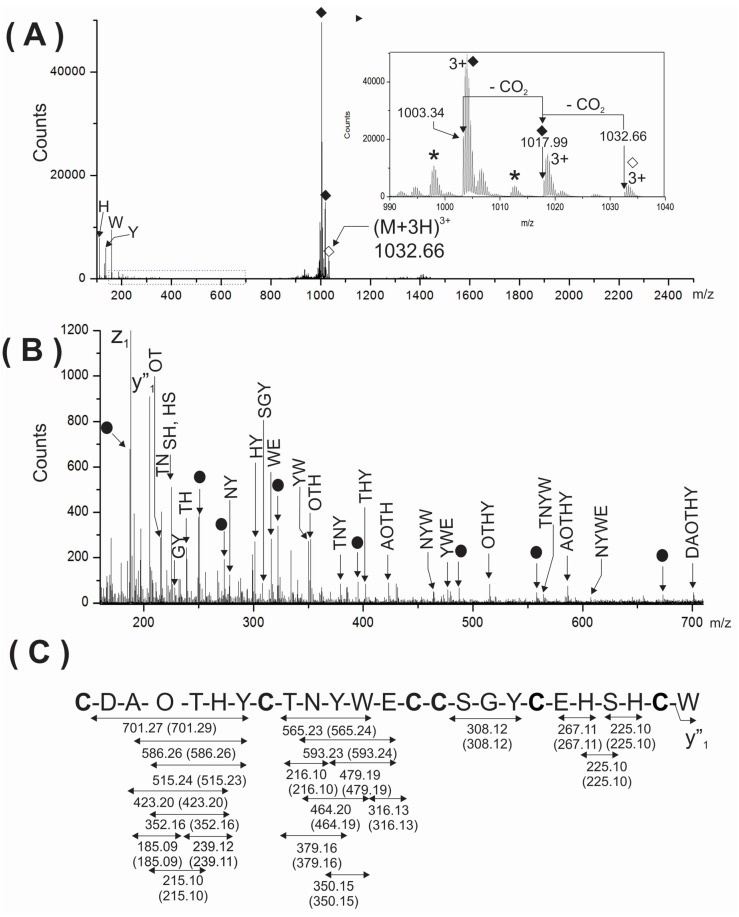
(**A**) MS/MS spectra of the (M + 3H)^3+^ ion at *m/z* 1033.32 of the native PiVIIA peptide. The inset at the right represents the region close to the precursor ion. Two consecutive losses of 44 Da were assigned to CO_2_ molecules from the precursor due to the presence of two γ-carboxyglutamic acids residues and are indicated with solid rhombuses. The asterisks represent losses of water. The precursor ion is indicated with an empty rhombus; (**B**) The expanded region (*m/z* 150–705) of the MS/MS spectrum shown in (**A**) is enriched in cysteine-free internal fragment ions. The filled circles correspond to Y*_n_*A*_m_* ions. The mass errors between the expected and experimental masses for all fragments ions were between 0.01 and 0.02 Da; (**C**) Summary of the internal fragment ions detected in (**B**). Calculated masses of internal ions are indicated between parentheses. O represents a hydroxyproline residue.

To further support the presence of two gamma-carboxy glutamic acids in the analyzed peptide, the triply-charged ion (*m/z* 1032.66) of the intact peptide was analyzed by ESI-MS/MS (Figure 4A). As expected, very few backbone fragment ions were detected due to the considerable structural constrains imposed by three disulfide linkages. This MS/MS spectrum showed the immonium ions corresponding to the aromatic amino acids (H, Y and W) present in the peptide and two very intense signals at *m/z* 1003.34 and 1017.99 (see solid rhombuses, inset Figure 4A) close to precursor ions. These signals were also assigned to two subsequent losses of two CO_2_ from the precursor ions. This behavior observed in CID experiments for the intact peptide, containing disulfide linkages and two gamma-carboxy glutamic acids are in agreement with the previous reports for conotoxins sharing similar characteristics [7,8]. Unexpectedly, the MS/MS spectrum of the intact peptide (Figure 4A) was also very useful and informative when the region from *m/z* 150–705 was magnified (Figure 4B). Segments of sequences comprised between cysteine residues were well represented in the MS/MS spectrum by several internal fragment ions devoid of cysteine. Their expected and experimental molecular masses are summarized in Figure 4C.

When this MS/MS spectrum (Figure 4B) was compared with the corresponding for the reduced and carbamidomethylated peptide (Figure 2A,B), the assignment of, backbone (b*_n_* and y*_n_*) and cysteine-containing internal fragment ions, was a more reliable and easier task. This comparison was a key element for a reliable *de novo* sequencing, of a region in the MS/MS spectrum difficult to interpret due to the coexistence of several fragment ions of different origins: N-, C-terminal ions, internal fragment ions as well and their associated ions originated by neutral losses (-NH_3_, -H_2_O, -CO_2_). Furthermore, several internal fragment ions comprised between ^1^Cys and ^8^Cys, were detected, and most of them contain the hydroxyproline residue at the fourth position. The high resolution and mass accuracy achieved in the MS/MS spectrum also supported the presence of hydroxyproline (113.047 Da) and avoided its misassignment as Leu/Ile (113.084 Da). See the good agreement obtained between the expected and experimental mass for internal fragment ions containing hydroxyproline (Figure 4C).

From the methodological point of view, the fragmentation of the intact peptide was very useful for the assignment of two gamma carboxyglutamic acids as well as for a more reliable interpretation of the MS/MS spectra of the reduced and *S*-alkylated peptide. To what extent the fragmentation of an intact conotoxin with several disulfide bonds could be a general approach that may contribute to a more reliable sequencing by mass spectrometry of the reduced and *S*-alkylated derivative will depend, of course, on the arrangement of cysteine residues.

At least in the present case, where the sequencing has been based mainly on mass spectrometry data, this approach permitted an unambiguous assignment of the internal fragment ions, and a more reliable sequencing because it avoids their wrong assignment as either N- or C-terminal ions.

To confirm the peptide sequencing depicted in Figure 2A,B, the reduced and S-alkylated peptide was digested with chymotrypsin. This digestion was useful because it separated into two chymotryptic peptides, the modified amino acids present in the original peptide (hydroxyproline and gamma-carboxy glutamic acids). A singly- and a triply-charged ions detected at *m/z* 862.30 (^1^<CDAOTHY^7^) and *m/z* 861.95 (^8^C*TNYWγC*C*SGYC*γHSHC*W^25^), respectively, were generated during this proteolytic digestion.

**Figure 5:**
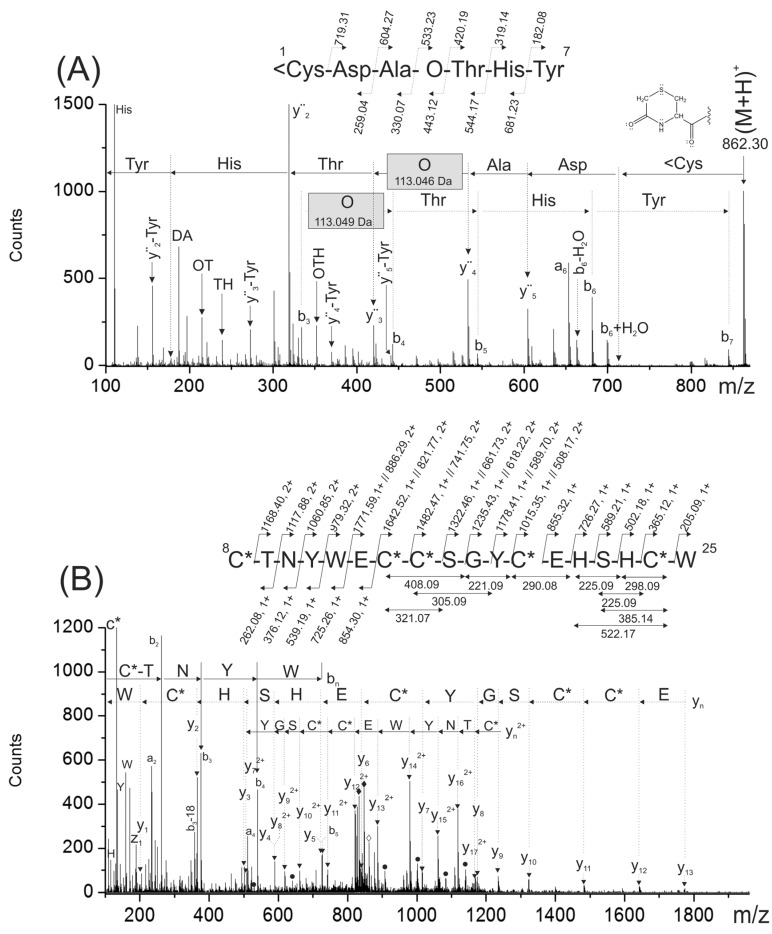
The ESI-MS/MS spectra shown in (**A**) and (**B**) correspond to singly- and triply-charged peptides ^1^<Cys-Tyr^7^ (*m/z* 862.30) and ^8^Cys-Trp^25^ (*m/z* 861.95) obtained from the chymotryptic digestion of the reduced and carbamidomethylated PiVIIA peptide. The gray rectangles indicate the mass difference assigned to the hydroxyproline residue. <C represents a cyclized cysteine residue located at the N-terminal end forming a cyclic of six-membered ring. In (**B**), the black rhombuses indicate triply-charged signals detected at *m/z* 847.28 and 832.62 that were assigned to two subsequent losses of CO_2_ molecules from the precursor. The filled circles correspond to y*_n_*^2+^ ions with masses increased by 44 Da due to the presence of a γ residue within their sequence.

The N-terminal chymotryptic peptide (^1^<CDAOTHY^7^) has a molecular mass 17 Da lower than expected. At the N-terminal end, it has a cyclic Cys residue forming a six membered-ring (<C), probably originated during proteolytic digestion due to the combined effect of a basic pH and temperature [10]. The analysis of the MS/MS spectra corresponding for this peptide (Figure 5A) confirmed the N-terminal sequence (Figure 2A,B). In this MS/MS spectrum, a C-terminal rearrangement ion (b_6_ + H_2_O) typical of peptides containing internal basic amino acids [11] was observed with appreciable intensity. Probably, the presence of a basic amino acid (His) located at *n* − 1 position make this rearrangement more favorable [12]. In addition, when this rearrangement ion (b_6_ + H_2_O) was further fragmented upon CID, several fragment ions devoid of the C-terminal Tyr were observed. These ions were assigned as y*_n_*-Tyr, (Figure 5A) and they were also useful to confirm the peptide sequencing.

The mass differences between consecutive backbone ions y_4_–y_3_ (113.049 Da) and b_4_–b_3_ (113.046 Da) confirmed the presence of a hydroxyproline residue (see gray rectangles in Figure 5A). The good agreement between the experimental masses of internal ions and backbone ions that contain hydroxyproline within their structures also confirmed the presence of this modified amino acid (data not shown). In this MS/MS spectra, signals previously assigned to CO_2_ losses from the precursor, were not observed since this fragment of PiVIIA (1–7) does not contain any gamma-carboxy glutamic acid.

On the other hand, the analysis of the MS/MS spectrum of the triply-charged chymotryptic peptide at *m/z* 861.95 (Figure 5B) confirmed the C-terminal sequence of the peptide. This MS/MS showed signals assigned to two CO_2_ losses from the precursor confirming by this way, the presence of two gamma-carboxy glutamic acids. In addition, y*_n_*^2+^ ion series with masses increased by 44 Da were observed (see filled circles in Figure 5B). Internal fragment ions were useful to confirm the C-terminal part of the sequence that was not verified simultaneously by the b*_n_* and y*_n_* ions.

The experimental molecular mass (*m/z* 861.95) and the calculated mass for the triple-charged ion of this peptide (*m/z* 861.95), considering the presence of two gamma-carboxy glutamic acids, agrees very well.

Although the Glutamic acids were detected in all ESI-MS/MS spectra as a free Glu residue (129.04 Da), the presence of two neutral losses of 44 Da from the precursor confirm that they are modified as γ residues.

### 2.4. Electrophysiological Characterization

A total of 30 DRG neurons (mean capacity = 44 ± 22 pF, Standard Deviation) were successfully voltage-clamped for a sufficient time to allow the study of PiVIIA actions. The mean capacitance of these neurons corresponds to a cell diameter of about 36 ± 9 μm. All voltage clamp experiments were performed using the whole-cell mode. In order to minimize the effects of time-dependent shifts in our results, recordings were not initiated until about 5–10 min after the whole-cell configuration was achieved, that is, when the signal had stabilized.

The Ca^2+^ currents were recorded applying a voltage ramp from −100 mV to +100 mV, with a duration of 500 ms, interval between sweeps was 10 s. Under control conditions, this stimulation protocol produced a current that activated around −35 mV reaching its maximum amplitude (1.7 ± 0.2 nA) around 8 mV, having a reversal potential around 45 mV. An estimated concentration of free Ca^2+^ is about 1.5 × 10^−9^ M in the pipette solution for the recording of Ca^2+^ current (0.1 mM Ca^2+^ and 10 mM EGTA). A calculated equilibrium potential for Ca^2+^ in this condition is about +180 mV, then the outward currents observed at voltages higher than +45 mV are most probably carried by Cs^+^ flow through voltage-gated Ca^2+^ channels. With perfusion of 3 μM PiVIIA in eight of 11 neurons, the peak amplitude increased by 29% ± 5% (*p* < 0.05), compared to the control. In the additional three neurons, a non-significant (*p* > 0.05) decrease in the peak amplitude of 6.8% ± 1.7% with respect to the control (data not shown) was observed.

**Figure 6:**
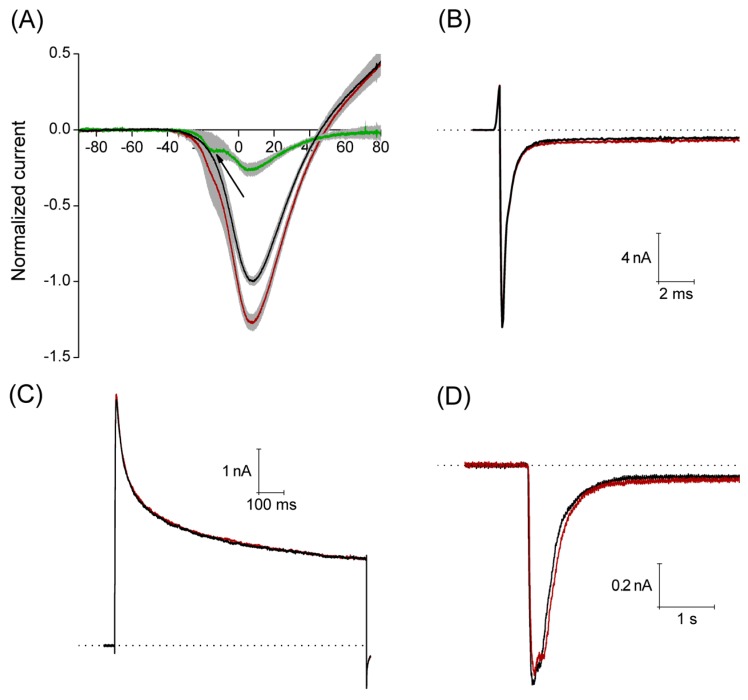
Peptide PiVIIA increases the amplitude of the calcium current without significantly modifying the Na^+^, K^+^ and ASIC currents. (**A**) Current-voltage relationship generated from a ramp pulse protocol. Solid lines represent the mean of eight experiments under control conditions (black line) and in the presence of 3 μM PiVIIA (red line). Shadows in light gray represent the standard error of the mean. Green trace depicts the PiVIIA-sensitive component of the Ca^2+^ current obtained by subtraction of the Ca^2+^ current in the presence of the toxin from that in the absence of the compound. Perfusion with PiVIIA revealed a low voltage activated component that probably corresponds to the T-type Ca^2+^ current (arrow); (**B**–**D**) Representative traces of sodium (**B**), potassium (**C**) and ASIC (**D**) currents in the absence (black lines) and presence (red lines) of PiVIIA. The dotted lines represent the zero current.

The maximum effect was reached within the first 2.5 min of the toxin perfusion, and it did not reverse after 2–5 min washout (Figure 6A). In the additional three neurons, a non-significant (*p* > 0.05) decrease in the peak amplitude of 6.8% ± 1.7% with respect to the control (data not shown) was observed. It is worth noting that the use of PiVIIA toxin reveals a low voltage activated component of the Ca^2+^ current that was not evident in the control condition (see arrow in Figure 6A), and that most probably corresponds to the T-type Ca^2+^ current (Ca_v_ 3.1–3.3).

With the aim of studying the selectivity of the toxin, in a set of experiments, we analyzed the actions of PiVIIA on sodium, potassium and acid sensing ionic channel (ASIC) currents. Sodium currents were evoked by a 40 ms step depolarization to a membrane potential of −10 mV from a holding potential of −100 mV, with an interval between sweeps of 8 s. The actions of PiVIIA were evaluated on:

(a) the maximum amplitude of the current (INa_max_),
(b) the time constant of the current inactivation (τ_h_), as derived from an exponential fit, and
(c) the relation between the current amplitude at the end of the voltage pulse and the peak current amplitude (INa_end_/INa_max_) which gives an estimate of the probability for the channels not to be inactivated at the end of the voltage pulse.

Perfusion of 10 μM PiVIIA (*n* = 5) for 2 min had no significant effect (*p* > 0.05) on any of the parameters studied: INa_max_ = 6.4 ± 2.8 nA *versus* 6.5 ± 2.7 nA; τ_h_ = 5.6 ± 1.9 ms *versus* 5.5 ± 1.8 ms; INa_end_/INa_max_ = 0.12 ± 0.09 *versus* 0.13 ± 0.1, under control conditions and in the presence of PiVIIA, respectively (Figure 6B).

Potassium currents were elicited by a single-step voltage protocol, where 1 s depolarizing pulses to 30 mV were applied from a holding potential of −90 mV every 8 s. The parameters measured were:

(a) the maximum amplitude IK_max_,
(b) the decay time constant τ, and
(c) the steady-state level (IK_ss_) of the current.

None of these parameters were significantly affected by the perfusion of 10 μM PiVIIA for about 2 min (*p* > 0.05, *n* = 5): IK_max_ = 4.1 ± 1.2 nA *versus* 4.0 ± 1.1 nA; τ = 308.3 ± 50.3 ms *versus* 329.4 ± 63.0 ms; IK_ss_ = 2.2 ± 0.7 nA *versus* 2.2 ± 0.7 nA, under control conditions and in the presence of PiVIIA, respectively (Figure 6C).

The ASIC currents were elicited by a fast (about 40 ms) pH change from 7.4 to 6.1 for 5 s, while keeping the cell at a holding potential of −60 mV. The interval between the pH change steps was 1 min to guarantee that the ASIC current was completely recovered from desensitization.

The parameters measured to characterize the action of PiVIIA on the ASIC currents were:

(a) Maximum peak amplitude (I_max_).
(b) Current desensitization time constant (τ_des_), determined by fitting the decay phase of the current with a single exponential function).
(c) The amplitude at the end of the 5 s acid pulse (I_ss_), which is a measure of the steady state current and it was calculated as the mean of the current in the last 200 ms of the acid pulse).

The compound was applied 20 s before the pH change to 6.1 and during the whole 5 s pH pulse. 3 μM PiVIIA did not produce any significant effect on the parameters studied (*p* > 0.05, *n* = 7): I_max_ = 1.1 ± 0.2 nA *versus* 1.0 ± 0.2 nA; τ_des_ = 343 ± 76 ms *versus* 358 ± 84 ms; I_ss_ = 0.04 ± 0.02 nA *versus* 0.05 ± 0.02 nA, under control conditions and in the presence of PiVIIA, respectively (Figure 6D).

## 3. Discussion

The biological activity assigned to γ-conotoxins is constrained to one study in isolated caudodorsal neurons from *Lymnaea* (a freshwater snail), where low concentrations (about 3 µM) of γ-PnVIIA produce depolarization, and larger doses (>10 µM) induce action potential discharge. Voltage clamp recordings showed that a non-specific cationic (Na^+^ or Ca^2+^) inward current was enhanced by the toxin [6].

In another work, the activity of γ-TxVIIA was studied in isolated medial neurons from the pleuropedal ganglia of *Aplysia* (a marine mollusk), and this peptide induced a depolarization and triggered action potential bursting [13]. Their similar biological effects in mollusks suggest similar molecular targets, or modulation of the same ionic current. Probably, they participate in the repetitive-pacemaker-activity on the nervous system of a mollusk, as part of a defensive mechanism.

In this work, it has been found that micromolar concentration of PiVIIA increase the magnitude of the macroscopic calcium current in DRG neurons from rat, without a significant modification of the Na^+^, K^+^ and ASIC currents. An increase, even modest of the calcium current, may have a significant impact in the excitability and electrical activity of neurons, and may account for the previous observations indicating that related γ-conotoxins trigger action potential bursting, setting up to PiVIIA as a member of the pharmacological family of the γ-conotoxins.

**Figure 7:**
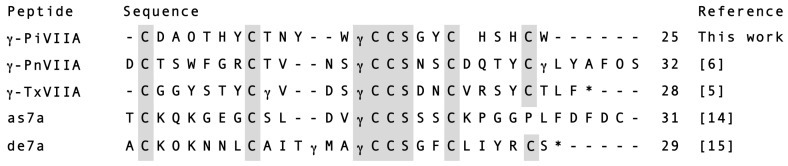
Alignment of conotoxin γ-PiVIIA with other gamma-conotoxins from *C. pennaceus* (γ-PnVIIA), *C. textile* (γ-TxVIIA), and with γ-conotoxin-like peptides from *C. austini* (as7a) and *C. delessertii* (de7a). For posttranslational modifications: O: 4-hydroxy proline; γ: γ-carboxyglutamate; *: amidated C-terminus. Identical residues with respect to γ-PiVIIA are shaded in gray. The numbers to the right indicate the sequence length of each conotoxin.

The arrangement of six Cys residues C-C-CC-C-C has been widely assigned to the O- conotoxins (*i.e.*, in members of the O1-, the O2-, and the O3-superfamily). Interestingly, the Cys spacing present in γ-PiVIIA (CX_6_-CX_5_-CCX_3_-CX_4_-C) is identical with γ-PnVIIA and γ-TxVIIA (Figure 7). Additionally, γ-PiVIIA shows 45% and 58% sequence identity with γ-PnVIIA and γ-TxVIIA, respectively.

Another characteristic that insert γ-PiVIIA into a γ-conotoxin member, is the unique structural motif, -γCCS- which is found in an extended form (-SγCCS-) in this family. The γ-conotoxin-like peptides as7a and de7a from the vermivorous species *C. austini* and *C. delessertii*, respectively, and the δ-conotoxin-like peptide de7b from *C. delessertii* did not have Ser in their motifs, showing only -γCCS- [14,15,16]. The latter motif is present in γ-PiVIIA, and was also isolated from a vermivorous species [17].

Biochemical parameters resulting from the comparison of γ-conotoxins and γ-conotoxin-like peptides showed that four of them (Table 1), share hydrophilic and acidic characteristics, being γ-PiVIIA the most hydrophilic one. Additionally, they have differential net charges and p*I*, that might be in principle related to the molecular target specificity and differences found in the biological activity. To unravel the structural-activity relationship of this peptide, further experiments should be performed.

**Table 1:** Biochemical parameters for comparison of all γ-conotoxins reported.

Conotoxin ^a^	Source ^b^	Hydropathicity ^c^	Arrangement ^d^	p*I* ^c^	Net Charge ^e^
γ-PiVIIA	V	−0.700 ^f^	CX_6_CX_5_CCX_3_CX_4_C	5.17 ^f^	−3 ^f^
γ-TxVIIA	M	−0.048 ^f^	CX_6_CX_5_CCX_3_CX_4_C	3.92 ^f^	−4 ^f^
γ-PnVIIA	M	−0.319 ^f^	CX_6_CX_5_CCX_3_CX_4_C	3.92 ^f^	−5 ^f^
as7a	V	−0.290 ^f^	CX_6_CX_5_CCX_3_CX_10_C	4.44 ^f^	−1 ^f^
de7a	V	+0.350 ^f^	CX_6_CX_7_CCX_3_CX_4_C	7.81 ^f^	0^f^

^a^: Names of γ-conotoxins were taken from the original references; ^b^: Feeding type of the species, where V is vermivorous and M is molluscivorous; ^c^: Calculated by means of the ProtParam program (web.expasy.org/protparam/) [18]: GRAVY, gran average of hydropathicity and isoelectric point (p*I*); ^d^: Cystein spacing arrangement; ^e^: Calculated values by the Innovagen program (pepcalc.com) [19]; ^f^: Approximate values, because post-translational modifications of residues are not taken into account by the Protprogram and the Innovagen program.

The effect of γ-PiVIIA was analyzed over Na^+^, K^+^ and ASIC currents based on the previous observation that suggested that toxin γ-PnVIIA might have actions on cationic channels permeable to Ca^2+^ and Na^+^ [6]. Regarding this, we studied two voltage dependent, and a chemically gated current (ASIC), in order to discern the selectivity of toxin γ-PiVIIA. In relation with the selectivity of γ-PiVIIA over the Ca^2+^ current subtypes, our data suggest that γ-PiVIIA potentiates both, the low voltage activated calcium current (Type-T: Ca_v_ 3.1–3.3) [20] and the high voltage activated calcium currents, most probably the N-type current (Ca_v_2.2). Type-T Ca^2+^ current in DRG neurons, has been shown to be mainly due to the expression of Ca_v_3.2 type T channels [21,22], and participates in setting the action potential threshold. The high voltage activated Ca^2+^ currents are mainly due to larger components carried by Ca_v_ 2.2 in these neurons [23], and this component of the Ca^2+^ current is mostly related to the neurotransmitter release. Although another factor that may account for the low voltage activated component of the current is that DRG neurons of the rat express TTX-resistant Na^+^ channels and, in the absence of Na^+^ in the extracellular solution, Ca^2+^ can pass through them. Therefore, the possibility that PiVIIA potentiates activity of TTX-R Na^+^ channels and causes appearance of the low-voltage activated currents cannot be completely excluded.

Both of these currents have been associated with nociception in animal models [24]. Thus, a toxin that affects them would constitute a useful tool for pain studies and the development of a pain management strategy. In addition, calcium currents and homeostasis of intracellular calcium have been shown to be upregulated in animal models of multiple sclerosis [25], and thus γ-PiVIIA may constitute an interesting molecular tool for simulating calcium channel upregulation in animal models.

## 4. Experimental Section

### 4.1. Venom Extraction of Conus Princeps

*Conus princeps* specimens were collected on the coastal region of Estacahuite bay in Oaxaca, Mexico. The venom ducts of five specimens were homogenized in 1 mL of 40% (*v*/*v*) ACN (Fermont Monterrey N. L., Mexico) containing 0.1% (*v*/*v*) TFA (Fluka, St. Louis, MO, USA). The homogenate was centrifuged at 10,000 ×*g* for 10 min at 4 °C. After centrifugation, the supernatant was separated, lyophilized, and stored at −80 °C for further experiments.

### 4.2. Purification of the Major Native Peptide

The lyophilized extract of the duct venom was solubilized into aqueous solution with 0.1% (*v*/*v*) TFA, was centrifuged at 10,000 × *g* for 5 min at 4 °C, and the supernatant was used for injection into a 1200 Series LC System (Agilent Technologies, Santa Clara, CA, USA). Deionized water was purified using a Milli-Q system (Pure Lab Flex, Elga from Ion Torrent, Life Technologies, Grand Island, NY, USA). The soluble venom was fractionated by means of RP-HPLC using an analytical C_18_ column (Zorbax 300SB; 4.6 mm × 250 mm, 5 μm particle size) with a pre-column (Zorbax 300-SB C18, 4.6 mm × 12.5 mm, 5 μm particle size) from Agilent Technologies. The column was equilibrated in a solution of 0.12% TFA (*v*/*v*). Fractions of the venom components were eluted with a linear gradient from 0 to 60% (*v*/*v*) of a ACN solution containing 0.12% (*v*/*v*) of TFA, over 60 min, at a flow rate of 1.09 mL·min^−1^. The major chromatographic fraction was repurified by RP-HPLC by using a linear gradient from 20% to 40% (*v*/*v*) of a ACN solution with 0.12% (*v*/*v*) TFA, over 40 min, at a flow rate of 0.5 mL·min^−1^. The absorbance was measured at 230 nm. Peptide was quantified on a UV-visible spectrophotometer NanoDrop 2000 (Thermo Scientific, Wilmington, DE, USA).

### 4.3. Reduction and S-Alkylation

The fraction containing the purified PiVIIA peptide (approximately 323 pmol) was dissolved in 10 μL of 1% (*w*/*v*), pH 8.3, ammonium bicarbonate buffer that contains 5 mM of dithiothreitol (DTT) and the solution was incubated for 2 h at 37 °C. The reaction was cooled at room temperature and an aqueous solution of iodoacetamide was added to reach a final concentration of 10 mM. The reaction proceeded for additional 30 min, in the darkness, and finally it was acidified by adding 10 μL of 0.1% (*v*/*v*) formic acid (FA) before liquid chromatography coupled to tandem mass spectrometry (LC-MS/MS) analysis. All reagents were obtained from Sigma-Aldrich (St. Louis, MO, USA).

### 4.4. Chymotryptic Digestion

The reduced and S-alkylated peptide were dissolved in 10 μL of 1% (*w*/*v*) ammonium bicarbonate buffer, pH 8.3 and one microgram of sequencing grade chymotrypsin (Sigma-Aldrich, St. Louis, MO, USA) was added. The proteolytic digestion proceeded during 4 h at 37 °C. The proteolysis was quenched by adding 5% (*v*/*v*) FA, and it was immediately analyzed by LC-MS/MS.

### 4.5. Automated Edman Degradation

The N-terminal sequencing was performed by taking an aliquot (~10 pmol) of the native peptide and dissolving it in 55 μL of 0.1% (*v*/*v*) TFA in 90% (*v*/*v*) aqueous ACN. Forty-five microliters were applied onto a glass fiber Micro TFA Filter (Applied Biosystems, Foster City, CA, USA) previously treated with 15 μL of Biobrene Plus (Applied Biosystems), dried with Argon, and then sequenced for 28 cycles in a Procise 491 Protein Sequencing System (Applied Biosystems) employing the pulsed-liquid method.

### 4.6. LC-MS and LC-MS/MS Analyses

The native peptide (96.9 pmol), as well as the S-alkylated peptide (40 pmol) and its chymotryptic fragments were loaded into an enrichment precolumn (40 nL, 5 μm) packed with Zorbax 300SB-C8 (300 Å) (SmMol-Chip 43 II, Agilent Technologies) that was previously equilibrated with 0.1% (*v*/*v*) TFA solution. The retained peptides were desalted by washing the precolumn with the same equilibration buffer during 5 min at a flow of 2 μL/min delivered by an Agilent 1260 Infinity capillary pump. After finishing the desalting step, the precolumn was online coupled to the analytical reversed phase column (75 mm, 43 μm) filled with the same packing material previously mentioned. The peptides were separated at a constant flow rate of 600 nL/min by mixing the buffer A (0.1% FA solution) and buffer B (90% of ACN/0.1% of FA) in a lineal gradient from 3% to 97% of B during 15 min by using two Agilent 1260 Infinity Nanoflow LC pumps.

The eluted peptides were sprayed into an G6530AA Accurate Mass quadrupole time-of-flight (QTOF) LC/MS System (Agilent Technologies) by using 2000 volts and 65 volts at the capillary and skimmer voltages, respectively. The high-resolution mass spectra (m/∆m~20,000) were acquired from *m/z* 300–2000 Da at 2 GHz, in only MS mode to detect the molecular mass of the peptides of interest. The mass spectrometer was calibrated using a mixture of politehylene glycols (Agilent Technologies).

The collision energies were customized for the individual precursor ions according their *m/z* and charge state to obtain high quality MS/MS spectra and perform a reliable *de novo* peptide sequencing. The MS/MS spectra corresponding to multiple-charged precursor ions were manually interpreted without the need of deconvolution to avoid the loss of low-intensity signals of relevance for *de novo* peptide sequencing. The data were acquired in positive ion mode and analyzed using the MassHunter Qualitative analysis software (Version 5.0 October 2011) from Agilent Technologies.

### 4.7. Dorsal Root Ganglion Neurons

Long–Evans rats CII/ZV at postnatal day 7–10 of either gender were used for the experiments. The handling of the animals was performed according to the “Guide for the Care and Use of Laboratory Animals” issued by the National Academy of Sciences and the “Regulations of the General Health Act” of the Health Secretary of Mexico. All efforts were made to minimize animal suffering and reduce the number of animals used. The DRG neurons were isolated from the vertebral column and incubated (30 min at 37 °C) in Leibovitz L15 medium (L15) (Invitrogen, Carlsbad, CA, USA) containing 1.25 mg/mL trypsin and 1.25 mg/mL collagenase (Sigma-Aldrich, St. Louis, MO, USA). After the enzymatic treatment, the ganglia were mechanically dissociated. The cells obtained were plated in 12 mm × 10 mm glass coverslips (Corning, Corning, NY, USA) pretreated with poly-d-lysine (Sigma-Aldrich) and placed onto 35 mm culture dishes (Corning). The DRG neurons were incubated 4–8 h in a humidified atmosphere (95% air, 5% CO_2_, at 37 °C) using a CO_2_ water-jacketed incubator (Nuaire, Plymouth, MN, USA) to allow them to settle and adhere to the coverslips. The incubation L-15 medium (pH 7.4) was added with 15.7 mM NaHCO_3_ (Merck, Naucalpan, Mexico), 10% fetal bovine serum, 2.5 μg/mL fungizone (both from Gibco, Waltham, MA, USA), 100 U/mL penicillin (Lakeside, Toluca, Mexico), and 15.8 mM HEPES (Sigma-Aldrich, St. Louis, MO, USA).

### 4.8. Electrophysiological Recording in DRG Neurons

A coverslip with attached cells was transferred to a 500 μL perfusion chamber mounted on the stage of an inverted phase-contrast microscope (TMS, Nikon Co., Tokyo, Japan). Current recording was made using an Axopatch 1D amplifier (Molecular Devices, Union City, CA, USA). All the experiments were made with a whole cell configuration (whole-cell patch clamp). Command-pulse generation and data sampling were controlled by pClamp 9.2 software (Molecular Devices) using a 16-bit data-acquisition system (Digidata 1320, Molecular Devices). Signals were low-pass filtered at 5 kHz and digitalized at 10 or 20 kHz, depending on the type of experiment. Patch pipettes were pulled from borosilicate glass capillaries (TW-120-3; World Precision Instruments, Sarasota, FL, USA) using a Flaming-Brown electrode puller (80-PC; Sutter Instruments Company, San Rafael, CA, USA). They had a resistance of 1.5 to 2.5 MΩ when filled with intracellular solution. The series resistances were compensated (≈80%). The seal and series resistances were continuously monitored in the time course of experiments, to guarantee stable recording conditions.

Solutions employed for electrophysiological experiments are depicted in Table 2 The recording chamber was perfused employing a peristaltic pump (Masterflex, L/S Easy-Load II, Cole Parmer Vernon Hills, IL, USA) that maintained the extracellular solution flowing at a rate of about 100 μL/min. Additionally the cells were continuously microperfused with extracellular solution or with extracellular solution plus toxin by using two square-tube rapid-solution changer (SF-778 Warner Inst., Hamden, CT, USA). All experiments were performed at room temperature (23–25 °C).

Ionic current data were analyzed off-line using Clampfit 10.2 (Molecular Devices) and Excel 2010 (Microsoft Co., Redmond, WA, USA). Statistical differences were determined using a Student’s *t* test with significance level of *p* < 0.05. Numerical data are presented as the mean ± standard error of the mean (SEM).

**Table 2:** Solutions used in the whole-cell clamp experiments. Concentration values are expressed in mM. Chol-Cl: Choline chloride; TEA-Cl: Tetraethylammonium chloride; 4-AP: 4-Aminopyridine; MES: (2(N-morpholino) ethanesulfonic acid, pK= 6.15) was used instead of HEPES (pK = 7.55) for working at pH 6.1. Intracellular solutions also contained 1 mM Na_2_GTP and 2 mM MgATP.

Solution	KCl	NaCl	CsCl	CdCl_2_	CsF	MgCl_2_	CaCl_2_	Chol-Cl	KF	HEPES	MES	EGTA	TEA-Cl	4-AP	pH
Extracellular I_Ca_^++^	-	-	5	-	-	-	2.5	-	-	10	-	-	130	10	7.4
Intracellular I_Ca_^++^	-	-	130	-	-	-	0.1	-		15		10	10	-	7.2
Extracellular I_Na_^+^	-	20	-	-	-	1	1.8	70	-	10	-	-	45	10	7.4
Intracellular I_Na_^+^	-	10	30	-	100	-	-	-	-	5	-	8	10	-	7.3
Extracellular I_K_^+^	10	-	-	0.3	-	1.2	1.8	130	-	10	-	-	-	-	7.4
Intracellular I_K_^+^	50	-	-	-	-	-	0.1	60	40	5	-	10	-	-	7.2
Extracellular ASICs pH 7.4	5.4	140	-	-	-	1.2	1.8	-	-	10	-	-	-	-	7.4
Extracellular ASICs pH 6.1	5.4	140	-	-	-	1.2	1.8	-	-	-	10	-	-	-	6.1
Intracellular ASICs	125	10	-	-	-	-	0.1	-	-	5	-	10	-	-	7.2

## 5. Conclusions

Our data clearly shows that toxin PiVIIA is a new member of the γ-conotoxin family that affects at least two subtypes of mammalian Ca^2+^ channels. Even when the previous representatives were characterized on molluscan pacemaker channels, all the γ-conotoxins described up to date upregulate voltage-gated cation currents, including γ-PiVIIA. This work opens up the possibility for discovering new relevant targets for γ-conotoxins in mammalian cells.
